# Association between Gut Microbiota Profiles, Dietary Intake, and Inflammatory Markers in Overweight and Obese Women

**DOI:** 10.3390/foods13162592

**Published:** 2024-08-19

**Authors:** Orada Chansa, Prapimporn Chattranukulchai Shantavasinkul, Wutarak Monsuwan, Jintana Sirivarasai

**Affiliations:** 1 Master of Science Program in Nutrition, Faculty of Medicine Ramathibodi Hospital, Institute of Nutrition, Mahidol University, Bangkok 10400, Thailand; orada.chansa@gmail.com; 2 Division of Nutrition and Biochemical Medicine, Department of Medicine, Faculty of Medicine Ramathibodi Hospital, Mahidol University, Bangkok 10400, Thailand; sprapimporn@gmail.com; 3 Nutrition Unit, Faculty of Medicine Ramathibodi Hospital, Mahidol University, Bangkok 10400, Thailand; wutarak.pue@mahidol.edu

**Keywords:** gut microbiome, macronutrients, inflammatory markers, obesity, dysbiosis

## Abstract

Being overweight and obesity are significant global public health challenges due to their association with adipose tissue dysfunction, pro-inflammatory marker production, and alterations in gut microbiota composition. To explore the relationship between gut microbiota, dietary factors, and inflammatory markers in overweight or obese women, we conducted a cross-sectional study involving a healthy group (*n* = 20) and an overweight or obese group (*n* = 75). We collected data, including clinical, anthropometric, and dietary assessments, and carried out a blood biochemical analysis, the measurement of inflammatory biomarkers (hs-CRP, IL-6, and TNF-α), and the 16S rRNA gene sequencing of fecal samples. The gut microbiota analysis revealed notable differences in alpha and beta diversity between the two groups. Moreover, the abundance of gut microbiota in the overweight or obese group correlated positively with adiposity markers, blood pressure, lipid profiles, and inflammatory markers. These findings highlight significant changes in gut microbiota associated with obesity, potentially implicating pathways such as lipopolysaccharide biosynthesis. Understanding the role of the gut microbiome in obesity could reveal specific avenues for intervention.

## 1. Introduction

Obesity is a condition that can trigger inflammation, dyslipidemia, insulin resistance, hypertension, and vascular endothelium dysfunction and is associated with serious health conditions such as cardiovascular diseases, non-alcoholic fatty liver disease, type 2 diabetes mellitus (T2DM), and cancers [1]. The causes of obesity are multifactorial, including excess energy consumption, low physical activity, and genetic, economic, social, psychological, and environmental factors [2]. Another significant factor is the gut microbiota, a community of microorganisms established since birth and influenced by dietary habits. These microorganisms impact immune responses, glucose metabolism regulation, and overall metabolic balance [3]. Low-grade inflammation in obesity is a consequence of gut microbiota alterations due to the presence of lipopolysaccharides (LPSs), which activate nuclear factor-κB (NF-κB) and interfere with the secretion of pro-inflammatory cytokines such as type I interferon (IFN)-γ, TNF-α, IL-6, IL-1β, IL-8, and MCP-1 [4,5]. Factors influencing gut bacteria include genetics, age, diet, antibacterial drugs, psychological conditions, exercise, and geographic location [6]. The composition and diversity of gut microbiota can be effectively altered by both short-term and long-term dietary changes, indicating that nutrition is a significant driver of microbial homeostasis [7]. Dietary factors impact the composition, function, and structure of the gut microbiota in numerous ways. Consumption of a high-caloric or high-fat diet may induce gut dysbiosis and inflammation, resulting in a leaky gut, while a diet rich in dietary fiber and trace elements or minerals can positively influence gut microbiota and intestinal health [8].

Previous studies exploring the association of gut microbiota with clinical variables in obese and lean participants have reported that several taxa, notably uncharacterized *Ruminococcaceae* and *Lachnospiraceae* UCG-010, were significantly lower in obese individuals and negatively correlated with body mass index (BMI), waist circumference (WC), and fat mass (FM). Conversely, higher levels of taxa such as *Acidaminococcus* and *Lachnospira* in the obese group showed positive associations with BMI, FM, and the waist-to-hip ratio (WHR). An exception was Akkermansia, which had a negative association with WHtR but was more prevalent in the obese group [9].

Similarly, another study identified associations between gut microbiota and inflammatory markers in a group of 86 obese individuals with an average BMI of 29.3 ± 4.1 kg/m^2^. These findings highlighted positive correlations between the abundance of *Bifidobacterium adolescentis* and *Alistipes onderdonkii* with IL-6 and high-sensitivity C-reactive protein (hs-CRP) levels, as well as *Eubacterium rectale* with hs-CRP levels [10]. Furthermore, significant findings emerged from a study involving 19 overweight or obese individuals who followed a calorie-restricted (20% to 40%), low-carb, high-fat diet for four weeks. This diet led to notable changes in the fecal microbiota, including an increase in bile-resistant bacteria such as *Enterobacteriaceae*, *Ruminococcus bicirculans*, *Butyricimonas*, and *Odoribacter splanchnicus*. Importantly, the decrease in BMI was directly associated with a reduction in bacteria that are often linked to inflammation, such as *Dorea* and *Collinsella* [11].

Based on previous studies, various factors can alter the composition of gut bacteria, leading to inflammation and metabolic issues, particularly in individuals with excessive fat accumulation. Additionally, there is limited research on the connection between gut bacteria, diet, and inflammation in Thai women. This gap in knowledge provides the basis for conducting a cross-sectional study to examine the gut bacteria profiles, dietary factors, and changes in inflammatory markers among overweight and obese women.

## 2. Materials and Methods

### 2.1. Study Participants

This cross-sectional analysis included 95 women aged 35–50 years, comprising 75 overweight or obese individuals and 20 participants with a healthy body weight. Sociodemographic, lifestyle, and medical history information was collected through standardized personal interviews. Participants provided details about their age, smoking status, frequency of alcohol consumption, education, occupation, and medical history. The exclusion criteria included the use of probiotics or recent antibiotic treatment within 4 weeks prior to enrollment, a history of cardiovascular disease, and medical treatment for conditions such as T2DM, dyslipidemia, hypertension, thyroid disease, liver and kidney diseases, cancer, or other chronic inflammatory diseases. Additionally, individuals with significant comorbidities, such as acute infections or chronic diseases, were excluded. The study was approved by the ethical committee of the Faculty of Medicine Ramathibodi Hospital, Mahidol University (COA.MURA2023/127 and 2023/791). The participants consented to participate in this study.

### 2.2. Anthropometric and Blood Pressure Measurement

Anthropometric measurements were taken by trained research assistants. Height was measured in meters and body weight in kilograms. WC was measured at the midpoint between the lowest rib and the iliac crest, and hip circumference was measured at the widest part of the hips. Blood pressure measurements were taken after participants had rested for 5 min using an automatic blood pressure device (OMRON, Mannheim, Germany) with a cuff appropriate for the upper arm width of each individual.

### 2.3. Dietary Intake Assessment

Participants recorded their dietary intake over 3 days, including 2 weekdays and one weekend day. The intake and amounts of foods consumed were confirmed through interviews using reference pictures of portion sizes. Nutrient intake was analyzed using the Thai food composition program INMUCAL-Nutrients, version 4.0 (2018), developed by the Institute of Nutrition, Mahidol University, Thailand. The analysis calculated nutrient intake such as energy, carbohydrates, fats, proteins (animal and vegetable), calcium, iron (Fe) in animal and vegetable, vitamin A, thiamine, riboflavin, vitamin C, and niacin.

### 2.4. Biochemical Analyses

Venous blood samples were collected after a twelve-hour fast. The serum or plasma obtained was promptly processed and used to measure the lipid profile (total cholesterol, triglyceride, high-density lipoprotein (HDL)-cholesterol, and low-density lipoprotein (LDL)-cholesterol), hs-CRP, blood urea nitrogen (BUN), creatinine, alkaline phosphatase (ALP), aspartate transferase (AST), alanine transaminase (ALT), insulin, fasting plasma glucose (FPG), and hemoglobin A1C (HbA1C) using standard laboratory methods on an automatic analyzer (Cobas-Mira; Roche, Milan, Italy). Homeostasis Model Assessment (HOMA)2 values were derived using the HOMA Calculator v2.2.3. Additionally, serum IL-6 and TNF-alpha concentrations were analyzed using the Human IL-6 Quantikine^®^ ELISA Kit (Cat. D6050 and Cat. DTA00D, R&D Systems, Minneapolis, MN, USA), following the manufacturer’s guidelines.

### 2.5. DNA Extraction, Sequencing, and Microbiome Data Analyses

In the study, each participant diligently collected a fecal sample using the provided fecal sample collection plasticware, which included a DNA/RNA-shield reagent. It is important to note that these collections occurred on the same day as their blood samples were taken. Subsequently, the specimens were carefully transported to the laboratory for further processing and analysis. During the de-identification process, special attention was given to the unique ID code assigned to each sample, ensuring that no identifiable names were associated with the samples in the records. This meticulous approach was crucial in maintaining the privacy and confidentiality of the participants involved in the study.

DNA was extracted from stool samples using the QIAamp Stool Mini Kit (Qiagen, United States). The quantity and quality of the DNA were assessed using a NanoDrop spectrophotometer and electrophoresis. Subsequently, the V4 hypervariable region of the 16S rRNA gene was amplified by PCR using 515F and 806R primers and a 2X KAPA hot-start ready mix. The PCR conditions included an initial denaturation at 94 °C for 3 min, followed by 25 cycles of 98 °C for 20 s, 55 °C for 30 s, 72 °C for 30 s, and a final extension step at 72 °C for 5 min. The 16S amplicons were then purified using AMPure XP beads and indexed using the Nextera XT index kit, followed by an additional 8 cycles with the same PCR conditions for library preparation. Finally, libraries were cleaned and pooled for cluster generation, and we performed 250-bp paired-end read sequencing on the Illumina^®^ MiSeq™.

A sequence analysis was conducted using QIIME 2 (version 2022.2). The raw sequence data were demultiplexed using the q2-demux plugin. Reads with expected errors (maxEE) higher than 3.0 were discarded using DADA2 (via q2-dada2). A phylogeny was constructed using the SEPP q2-plugin, which placed short sequences into the sepp-refs-gg-13-8.qza reference phylogenetic tree. Alpha and beta diversity metrics and a Principal Coordinate Analysis (PCoA) were estimated using q2-diversity after the samples were rarefied (subsampled without replacement) to a minimum read count. Taxonomy was assigned using the classify-sklearn naïve Bayes taxonomy classifier against the SILVA (version 138.1) 99% operational taxonomic units (OTUs) reference sequences. A correlation heatmap visualization was performed using the ggplot2 package of the Python module plotnine.

Microbiome data underwent statistical analyses to assess alpha and beta diversity using the Kruskal–Wallis test and permutational multivariate analyses of variance (PERMANOVA) with 999 permutations, respectively. Differential abundance analyses were conducted using LEfSe, involving nonparametric factorial Kruskal–Wallis tests followed by a linear discriminant analysis and 30-fold bootstrapping (cutoff = logarithmic LDA score of ≥2.0). *p*-values were adjusted for multiple hypothesis testing using the Benjamini and Hochberg false discovery rate correction. Additionally, the MaAsLin2 R package was used for multivariable association analysis between experimental metadata and microbial features, controlling for fixed effects such as BMI and age.

### 2.6. Statistical Analysis

The statistical analysis was performed using SPSS 23 for Windows (IBM Corp., Armonk, NY, USA). A significance level of *p* < 0.05 was set for all analyses. Data normality was tested using the Kolmogorov–Smirnov test. Descriptive statistics were reported as medians with ranges or numbers with percentages. The Mann–Whitney U test was used to compare non-normally distributed continuous data between two groups. Spearman’s correlation coefficient was used to test correlations between inflammatory markers, various biochemical and anthropometric parameters, and dietary intake.

## 3. Results

### 3.1. Clinical Characteristics of Study Participants

This study included 95 women, divided into two groups: healthy (*n* = 20) and overweight or obese (*n* = 75), with median ages of 38 and 41 years, respectively. There were significant differences in blood pressure, WC, HC, WHR, FM, and the percentage of body fat between the two groups (all *p* < 0.05). The overweight or obese group also showed significantly higher levels of lipid profile indicators (total cholesterol, triglycerides, and LDL-cholesterol), except for HDL-cholesterol, as well as abnormal hyperglycemia markers such as FPG, HbA1C, insulin, and the Homeostatic Model Assessment for Insulin Resistance (HOMA-IR) compared to the healthy group. Inflammatory markers associated with obesity, including hs-CRP, IL-6, and TNF-alpha, also demonstrated significantly higher levels in the overweight or obese group (Table 1).

### 3.2. Comparison of Nutrient Intake in the Overweight or Obese and Healthy Groups

The nutrient intake data from a 3-day period are summarized in Table 2. There was no significant difference in total calorie intake between the overweight or obese and healthy subjects. However, the overweight or obese group showed higher percentages of carbohydrate and protein intake relative to total energy compared to the healthy group (all *p* < 0.05). Conversely, the fat intake and the percentage of energy from fat were lower in the overweight or obese group than in the healthy group. The overweight or obese participants also exhibited a tendency for higher intake of minerals, trace elements, and vitamins compared to those with a healthy weight. Notably, significant levels of calcium, iron (from animal sources), and sodium were observed in the overweight or obese subjects, while magnesium intake was lower in the healthy group. Additionally, the overweight or obese group had a lower intake of zinc and beta-carotene compared to the healthy group.

### 3.3. Association between Inflammatory Markers and Clinical, Biochemical, and Dietary Variables

The data revealed significant positive associations between the inflammatory markers (hs-CRP, IL-6, and IL-10) and systolic blood pressure (SBP), diastolic blood pressure (DBP), BMI, WC, HC, WHR, FM, or body fat percentage (all *p* < 0.05) (Table 3). Biochemically, there were statistical correlations observed between hs-CRP and lipid profile, FPG, insulin, HOMA-IR, AST, or ALT. Additionally, an increase in IL-6 levels was associated with LDL-C, insulin, or HOMA-IR, whereas TNF-α levels exhibited positive correlations with TC, TG, LDL-C, insulin, or HOMA-IR (all *p* < 0.05). A further analysis with dietary intake data indicated a positive association between hs-CRP levels and total calories, fat intake, percentage of energy from fat, or cholesterol intake, while IL-6 levels showed significant increases with increases in the percentage of energy from fat.

### 3.4. Alpha and Beta Diversity

**Figure 1:**
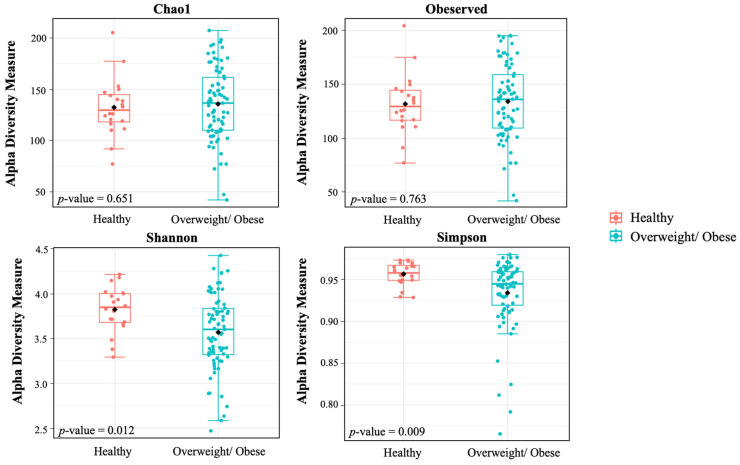
Revised Y axis and format of *p*-value Alpha diversity of the healthy and overweight/obese group. The alpha diversity represented within-sample diversity.

**Figure 2:**
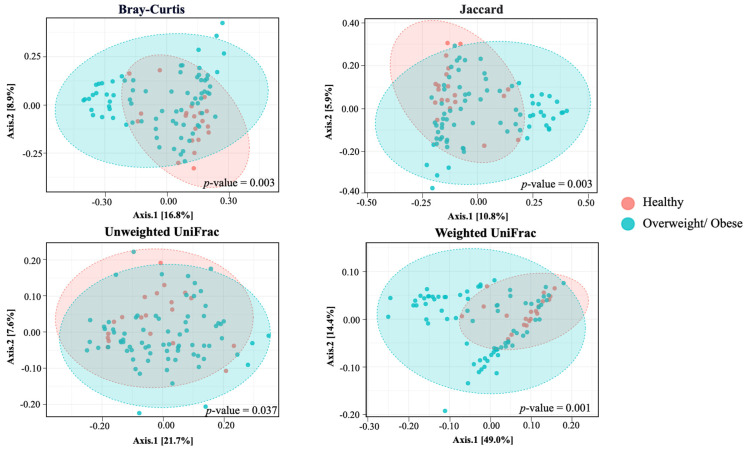
Revised the format of *p*-value Beta diversity of the healthy and overweight/obese group. Beta diversity represented the difference in species diversity between groups. The percent variation explained by each axis is in parenthesis, calculated from the PCoA eigen values.

We employed four metrics to analyze alpha diversity: the observed species, ChaoI index, Shannon index, and Simpson index, all of which measure richness and evenness. Our findings revealed significantly lower richness in alpha diversity indices, specifically in the Shannon (*p* = 0.012) and Simpson indexes (*p* = 0.009), in the overweight or obese group compared to the healthy group (Figure 1). The PCoA based on non-phylogenetic (Bray–Curtis dissimilarity, Jaccard distance) and phylogenetic methods (UniFrac distance) displayed a distinct separation between the gut microbiota community of the overweight or obese and healthy groups, a result confirmed by the pairwise PERMANOVA analysis. These outcomes demonstrate a significant difference in beta diversity between the two groups (Figure 2) (*p* = 0.003 for Bray–Curtis, *p* = 0.003 for Jaccard, *p* = 0.037 for unweighted UniFrac distance, and *p* = 0.001 for weighted UniFrac distance).

### 3.5. Differences in Bacterial Composition between the Overweight or Obese and Healthy Groups

**Figure 3:**
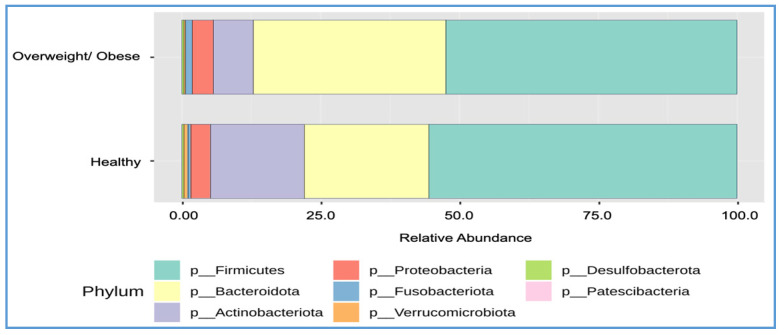

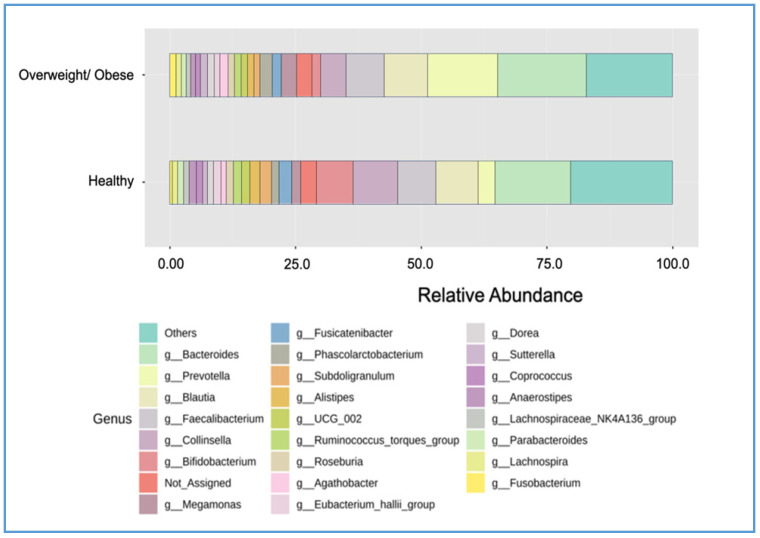
The relative abundances of family and genus between the healthy and overweight/obese groups.

The gut microbiota composition analysis at the phylum level revealed that *Firmicutes* accounted for 52.5% in overweight or obese individuals and 55.6% in healthy individuals, while Bacteroidetes comprised 34.6% in overweight or obese individuals and 22.4% in healthy individuals (Figure 3 and Table 4). The overweight or obese group exhibited significantly higher levels of Bacteroidetes (34.6% vs. 22.4% in the healthy group) and significantly lower levels of Actinobacteriota (7.3% vs. 17.0% in the healthy group). No significant differences were observed in the relative abundance of *Firmicutes*, *Proteobacteria*, *Verrucomicrobiota*, *Fusobacteriota*, or *Desulfobacterota* between the two groups. The *Firmicutes* to *Bacteroidetes* (F/B) ratio was 1.96 in the overweight or obese group and 2.80 in the healthy group, with a statistically significant difference (*p* = 0.035).

Further analysis at the genus level identified 24 major genera that showed significant differences between the overweight or obese and healthy groups (Table 5). Among the 13 genera more prevalent in the overweight or obese group were those from different phyla such as *Firmicutes* (*Ruminococcus gnavus*, *Lactococcus*, *Sellimonas*, *Lachnospiraceae* UCG-004, *Butyricicoccus*, *Lachnospiraceae* UCG-008, *Holdemanella*, *Dialister*, and *Megamonas*), Bacteroidota (*Prevotella* and *Bacteroides*), and *Pseudomonadota* (*Sutterella*) (all *p* < 0.05). Conversely, 11 genera were less prevalent in the overweight or obese group, including *Acidaminococcus*, *Coprobacillus*, *Lactobacillus*, *CAG_352*, *Megasphaera*, *Eubacterium ruminantium group*, and *Faecalibacterium* from the *Firmicutes* phylum; *Bifidobacterium*, *Collinsella*, *Senegalimassilia* from the *Actinomycetota* phylum; and *Akkermansia* from the *Actinomycetota* phylum (all *p* < 0.05).

**Figure 4:**
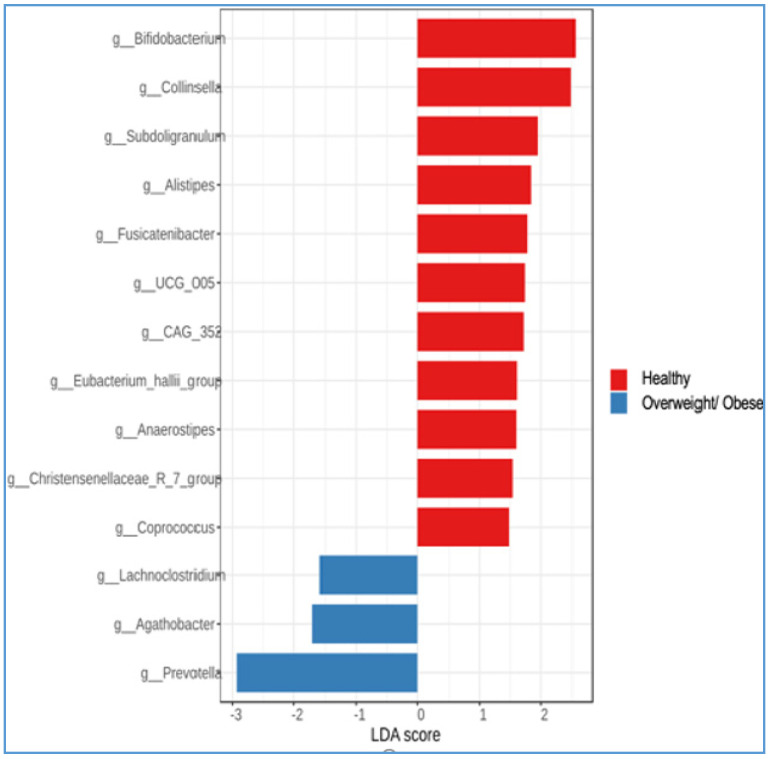
The results of Linear Discriminant Analysis Effect Size (LEfSe). The bar graph of LDA scores showed the taxa with the statistical differences between the two groups.

The results of the Linear Discriminant Analysis Effect Size (LEfSe) revealed distinct microbial profiles between the groups: 13 genera in the healthy group vs. 3 in the overweight or obese group (Figure 4). Notably, the LDA scores (log10) greater than two for the overweight or obese group included *Prevotella*, *Lachnoclostridium*, and *Agathobacter*. In contrast, the healthy group had higher LDA scores for *Bifidobacterium*, *Collinsella*, and others.

### 3.6. Associations between Anthropometric, Biochemical, Inflammatory, and Dietary Parameters and the Gut Microbiota (at the Genus Level)

**Figure 5:**
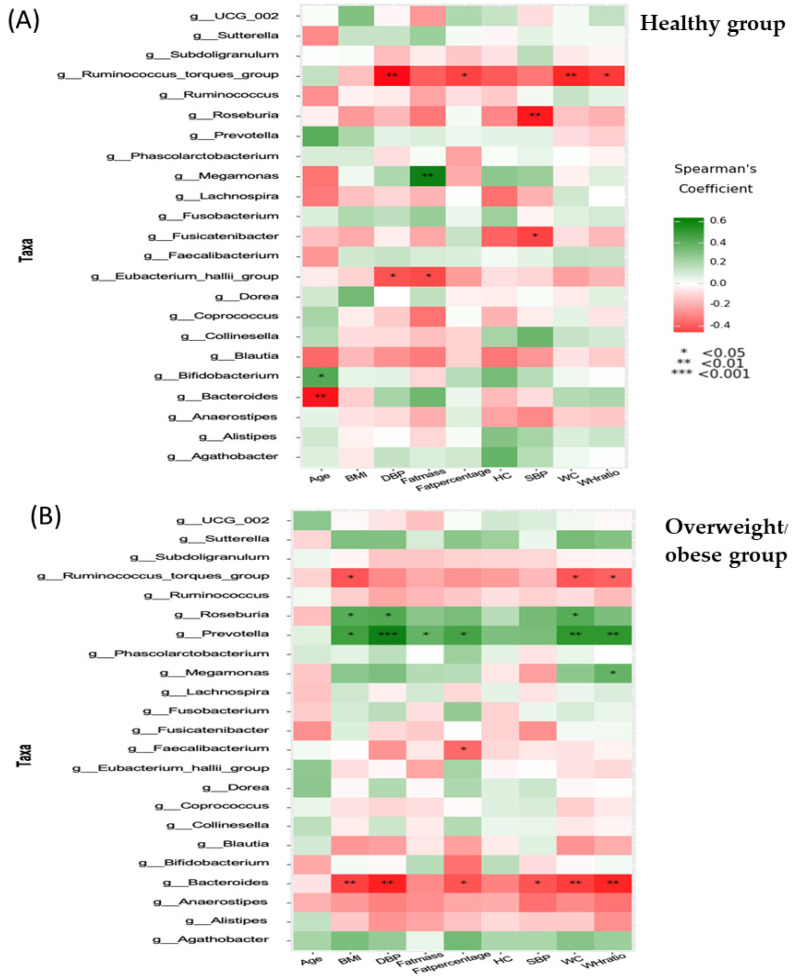
Correlation between age, blood pressure, and anthropometry and microbiota in the healthy group (**A**) and the overweight/obese group (**B**).

**Figure 6:**
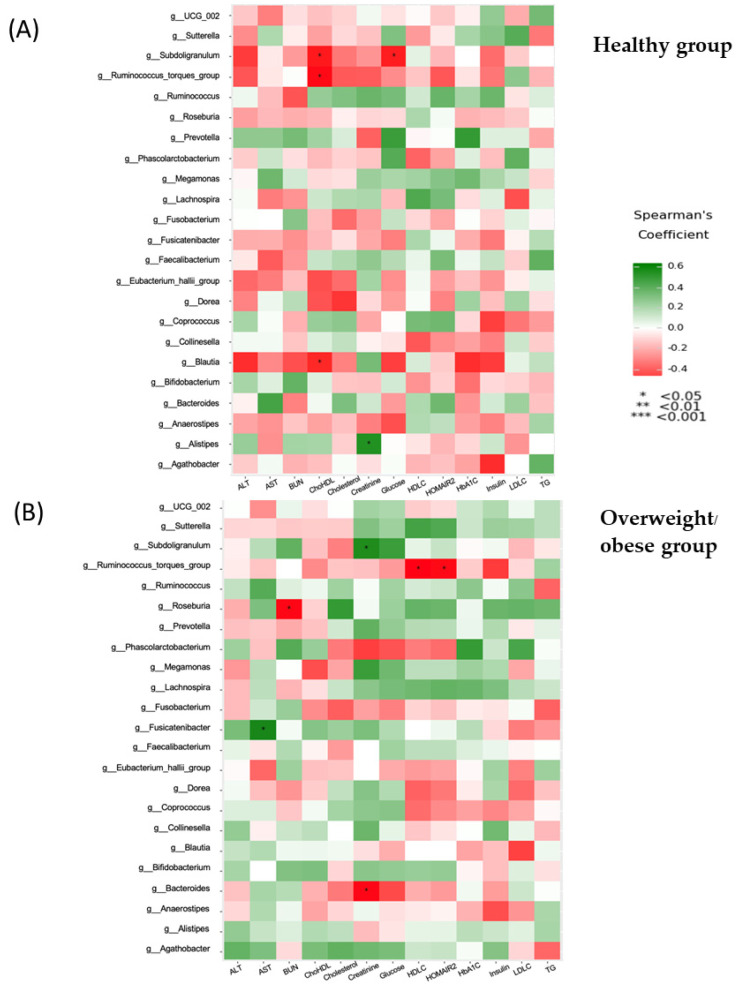
Correlation between biochemical parameters and microbiota in the healthy group (**A**) and the overweight/obese group (**B**).

**Figure 7:**
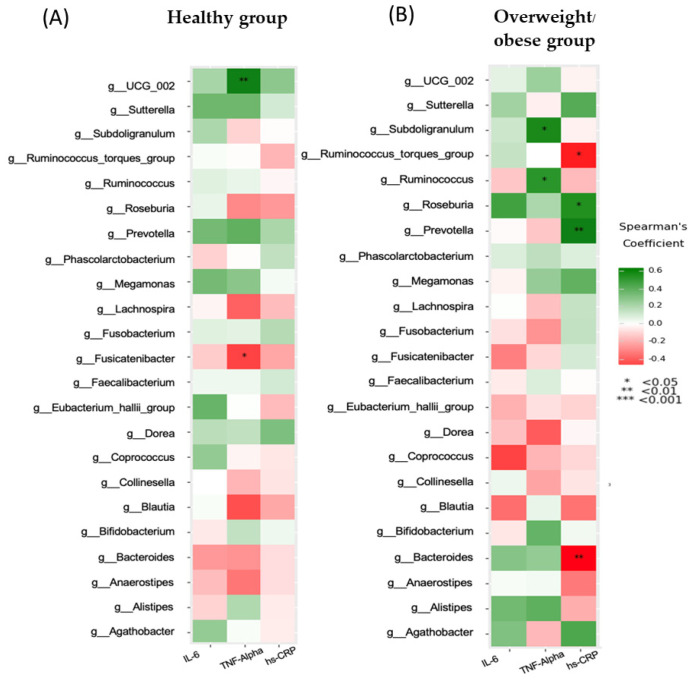
Correlation between inflammatory markers and microbiota in the healthy group (**A**) and the overweight/obese group (**B**).

**Figure 8:**
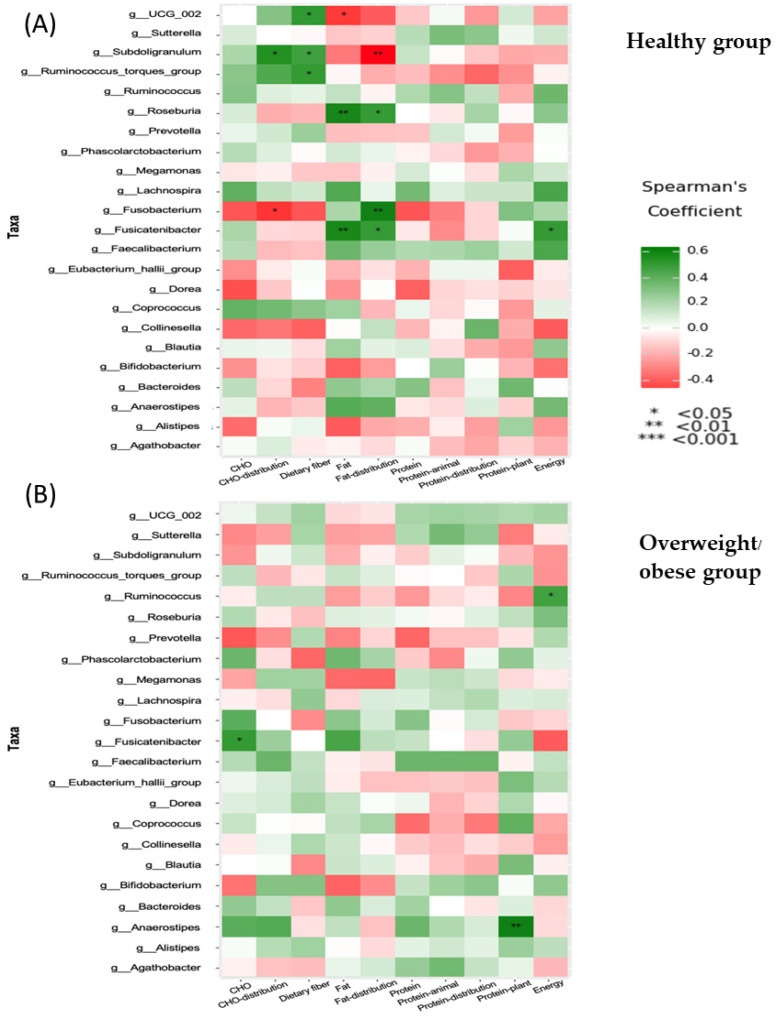
Correlation between dietary intake and microbiota in the healthy group (**A**) and the overweight/obese group (**B**).

A Spearman correlation analysis was performed to assess associations between gut microbiota and various parameters, using the ggplot2 package of the Python module plotnine (Figure 5, Figure 6, Figure 7 and Figure 8). In the healthy group, we observed several significant correlations. We identified positive associations between *Megamonas* and FM, and between *Bifidobacterium* and age. Conversely, we found negative associations between the *Ruminococcus torques* group and DBP, body fat percentage, WC, or WHR, and between *Roseburia* or *Fusicatenibacter* and SBP. Additionally, the *Eubacterium hallii* group correlated negatively with DBP or FM, and *Bacteroides* correlated negatively with age (Figure 5A).

In the overweight or obese group, we found a negative correlation between the *Ruminococcus torques* group and BMI, WC, or WHR; *Faecalibacterium* and body fat percentage; and *Bacteroides* and SBP, DBP, body fat percentage, BMI, WC, or WHR (Figure 5B). There was a significant positive correlation between *Roseburia* and DBP, BMI, or WC; *Prevotella* and DBP, BMI, FM, body fat percentage, WC, or WHR; and *Megamonas* and WHR.

Correlations between biochemical parameters and different genera between healthy and overweight or obese groups are presented in Figure 6A,B. In the healthy group, *Subdoligranulum* displayed negative associations with the cholesterol–HDL-C ratio and FBG, while the *Ruminococcus torques* group and *Blautia* also had negative correlations with the cholesterol–HDL-C ratio. Conversely, *Alistipes* exhibited a positive correlation with blood creatinine (Figure 6A). Notably, there were significant positive correlations between *Subdoligranulum* and creatinine and between Fusicatenibacter and AST levels, whereas negative correlations were observed between the *Ruminococcus torques* group and HDL-C or HOMA-IR; *Roseburia* and blood BUN; and *Bacteroides* and creatinine (Figure 6B).

Furthermore, inflammatory markers in the healthy group showed a significant positive correlation between UCG-002 and TNF-alpha levels and a negative correlation between *Fusicatenibacter* and TNF-alpha levels (Figure 7A). Meanwhile, TNF-alpha levels in the overweight or obese group showed positive correlations with *Subdoligranulum* and *Ruminococcus*. Another inflammatory marker, hs-CRP levels, was positively associated with *Roseburia* or *Prevotella* and negatively associated with the *Ruminococcus torques* group or *Bacteroides* (Figure 7B).

This study analyzed the nutrient intake and gut microbiota composition of both healthy and overweight or obese groups of people. Data from three-day food records were examined for energy and macronutrients, minerals, trace elements, and vitamins. In the healthy group, positive correlations were found between certain gut microbiota (UCG-002, *Subdoligranulum*, and the *Ruminococcus torques* group) and dietary factors. Moreover, positive correlations were observed between *Subdoligranulum* and dietary fiber or carbohydrate (CHO) percentage distribution; *Roseburia* and fat intake or percent fat distribution; and *Fusicatenibacter* and fat intake, percent fat distribution, or total energy. Additionally, negative correlations were found between UCG-002 and dietary fiber; *Subdoligranulum* and percent fat distribution; and *Fusobacterium* and percent CHO distribution (Figure 8A). For the overweight or obese group, positive correlations were observed between *Ruminococcus* and total energy, *Fusicatenibacter* and carbohydrate intake, and *Anaerostipes* and plant-protein intake (Figure 8B).

### 3.7. Functional Differences of Gut Microbiota in the Healthy and Overweight or Obese Groups

**Figure 9:**
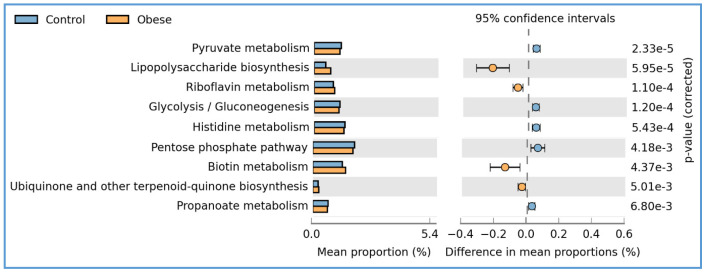
Comparison of PICRUSt predicted KEGG function data. An extended error bar plot for the comparison of healthy vs. overweight/obese groups. (Only functions with *p* < 0.05 are shown).

Our study involved a comprehensive exploration of the metabolic pathways within the gut microbiota, aiming to understand their association with the metabolic disparities observed in the healthy and overweight groups. This was accomplished through a rigorous comparative prediction analysis of the functional metagenome (PICRUSt) of the gut bacterial microbiota (Figure 9). The analysis led to the identification of nine metabolic pathways in which the difference in the percentage of relative frequency was statistically significant among the two groups (all *p* ≤ 0.01). In the healthy group, pathways such as pyruvate metabolism, glycolysis or gluconeogenesis, histidine metabolism, the pentose phosphate pathway, and propanoate metabolism (CHO metabolism) were enriched. Conversely, the overweight or obese group showed enriched levels of LPS biosynthesis, riboflavin metabolism, biotin metabolism, and ubiquinone and other terpenoid-quinone biosynthesis.

## 4. Discussion

This study demonstrated that the overweight or obese group showed a significant correlation between adiposity and WC, HC, or WHR, similar to a notable association between WHR and metabolic syndrome in obese adolescents [12] and middle-age Chinese individuals [13]. Understanding the possible mechanisms behind adiposity-induced metabolic consequences is key to addressing this health issue. An increase in adiposity is generally associated with an atherogenic lipid profile, including elevated blood triglyceride and LDL-C levels, reduced blood HDL-C levels, and the adiposopathic metabolic consequences of obesity such as high blood glucose or blood pressure levels [14]. These metabolic consequences were also observed in the overweight or obese individuals in our study. To assess the association between chronic low-grade inflammation and obesity, we measured the levels of hs-CRP, IL-6, and TNF-alpha. The results were consistent with significantly elevated levels of TNF-alpha identified in a cross-sectional study comprising 117 obese (BMI ≥ 30) and 83 non-obese, community-based volunteers [15], and a longitudinal study that showed a significant difference in hs-CRP values across all obesity categories [16]. Fat accumulation, especially around the abdomen, is strongly associated with the development of adipocyte hyperplasia and hypertrophy, leading to cytokine production such as TNF, IL-6, IL-1, IL-18, and chemokines. Additionally, CRP transcription predominantly occurs in hepatocytes in response to heightened cytokine levels, particularly IL-6 [17].

In this study, significant differences in carbohydrate, protein, and fat intake between the healthy and overweight or obese groups were reported. The result from a previous study indicated that total fat was related to a higher risk of obesity, whereas a high carbohydrate intake was related to a lower risk of obesity in women [18], which contrasts with another finding in which no differences in total energy and macronutrient intakes were shown [19]. Discrepancies in findings may be due to sample size, assessment methods, and participants’ dietary habits. Additionally, previous studies suggested that individuals who are obese tend to under report their daily food intake [20].

Our results showed an association between increasing serum hs-CRP levels and increasing cholesterol intake, similar to various reports [21,22]. These findings may be supported by the direct effect of a high-fat diet on adipogenesis, causing the dysfunction of adipose tissue and an increase in CRP and pro-inflammatory cytokines (IL-6 and TNF-α) production, or by the indirect effect of a high fat intake triggering LPS and up-regulating IL-6 and TNF-α [23].

Dysbiosis of the gut microbiota has been correlated with obesity, contributing to its development and progression by increasing energy absorption, influencing appetite, promoting fat storage, causing chronic inflammation, and leading to various metabolic disorders [24]. The normal human gut microbiota is mainly composed of *Firmicutes*, *Bacteroidetes*, *Proteobacteria*, *Actinobacteria*, *Fusobacteria*, and *Verrucomicrobia*, with *Firmicutes* and *Bacteroidetes* being dominant [25], similar to our findings (Table 4). Alpha diversity was significantly lower in the overweight or obese group compared to the healthy group, and beta diversity showed significant differences between the groups, consistent with available data. A lower alpha diversity (Shannon index) in obese versus non-obese adults was observed in 9 out of 22 studies, while a meta-analysis of seven studies revealed a non-significant mean difference [26]. Additionally, we observed a significantly lower F–B ratio in the overweight or obese individuals compared to the healthy group (Table 4). Conversely, a meta-analysis indicated a higher F–B ratio in obese individuals [26]. Several studies suggest that varying proportions of *Bacteroidetes* and *Firmicutes* in obese and non-obese individuals could be attributed to circulating metabolites, particularly short-chain fatty acids (SCFAs) [27]. Other influencing factors include methodological differences in sample processing, DNA sequence analysis, and the inadequate characterization of recruited participants, particularly the lack of consideration of lifestyle-associated factors [28].

The reasons behind changes in the complex gastrointestinal microbiome ecosystem due to obesity remain controversial. LEfSe analysis revealed that the more prevalent genera in the overweight or obese group were *Lachnoclostridium*, *Agathobacter*, and *Prevotella*, while the healthy group had a higher abundance of *Bifidobacterium*, *Collinsella*, *Subdoligranulum*, and *Alistipes* (Figure 4). A cross-sectional study in Emirati participants identified differences in taxa using LEfSe, with the obese group showing three key genera: two from *Firmicutes* (*Lachnospira* and *Acidaminococcus*) and one from *Verrucomicrobia* (*Akkermansia*) [9]. It is more likely that differences at the microbiota’s genus level between the overweight or obese and healthy groups were observed in our results, including high abundances in the healthy group of *Lactobacillus*, *Faecalibacterium*, *Coprobacillus*, etc. (Table 5). Interestingly, previous studies revealed both elevated and reduced levels of *Lactobacillus* related to obesity [29,30].

*Bifidobacterium*, *Collinsella*, and *Senegalimassilia* (belonging to the *Actinomycetota* phylum) were significantly higher in the healthy group. Similar to previous findings, the median level of *Bifidobacterium* in visceral obesity was 4.78, compared to 5.36 in the lean group, with the difference being statistically significant (*p* < 0.05) [31]. Additionally, a cross-sectional study in 96 overweight or obese subjects and 32 lean participants found that *Collinsella* could serve as a potential biomarker for obesity [32]. Significant increases in microbiota abundance in the overweight or obese group were reported for various genera, including *Bacteroides* and *Prevotella* (belonging to the *Bacteroidota* phylum), *Sutterella* (in the *Pseudomonadota* phylum), and *Megamonas*, *Lachnoclostridium*, and *Lachnospiraceae* (belonging to the *Firmicutes* phylum) (Table 5). A significant difference in *Prevotella* abundance was observed between the obesity group (30.57%) and healthy Chinese volunteers with a mean BMI of 20.2 kg/m^2^ (7.22%) [33]. Furthermore, Hu et al. (2015) reported significantly higher populations of *Bacteroides* and *Prevotella* in the obese group compared to those with a normal BMI, with both genera showing the most significant associations with BMI [34]. Next, we sought to understand which gut microbial taxa and functions were correlated with clinical, biochemical, and inflammatory parameters and dietary factors in both study groups (Figure 5, Figure 6, Figure 7 and Figure 8). In our study, we found a negative correlation between the *Ruminococcus torques group* and fat accumulation, contrasting with a previous study that reported a strong correlation between *Ruminococcus torques* and visceral fat area [35].

We observed that *Roseburia* and *Prevotella* were both positively correlated with BMI and adiposity, while Bacteroides showed a negative correlation with BMI and adiposity in the overweight or obese group. *Roseburia’s* increased abundance in individuals with an elevated BMI may be due to its ability to break down polysaccharides into SCFAs, leading to greater energy extraction from the diet [36,37]. *Prevotella* plays a role in activating the inflammatory response and is linked to elevated levels of circulating succinate in obese individuals, affecting glucose metabolism [38,39]. *Bacteroides* produces propionate, which reduces body weight gain and adiposity independently of food intake [40]. In the healthy group, the *Subdoligranulum* genus was negatively correlated with the total cholesterol to HDL-C ratio, and various metabolic risk parameters in the overweight or obese group showed that TNF-α levels correlated with both *Subdoligranulum* and *Ruminococcus*, while hs-CRP had a positive correlation with both *Roseburia* and *Prevotella*. Notably, *Prevotella* exhibited a positive correlation with hs-CRP and showed a significant increase in individuals with obesity, indicating potential implications for systemic disease outcomes [41].

Significant associations between various genera of microbiota and energy, CHO, protein, fat, and dietary fiber intake were identified in our results (Figure 8). We found significant associations between *Ruminococcaceae UCG-002*, the *Ruminococcus torques* group, or *Subdoligranulum* and a high fiber intake. A previous study revealed that resistant starch-enriched wheat or whole-grain wheat induced increases in the *Ruminococcus* genus [42]. *Fusicatenibacter* and *Roseburia* from the Lachnospiraceae family correlated with fat intake and distribution in the healthy group, potentially leading to low-grade inflammation through LPS translocation [43].

We employed a PICRUSt analysis to examine the impact of microbial abundance on metabolic pathways in the healthy and overweight or obese groups. Our findings revealed increased activity in specific metabolic pathways in the overweight or obese groups, including LPS biosynthesis, riboflavin metabolism, biotin metabolism, and ubiquinone and other terpenoid-quinone biosynthesis (Figure 9). This is consistent with previous research indicating that obesity resulting from diet can alter gut microbiota, leading to elevated intestinal permeability and higher levels of pro-inflammatory bacterial products such as LPS [44]. These changes can influence adipose tissue function, impair fat cell function, and increase the risk of obesity-related diseases [45]. Additionally, disturbances in biotin and ubiquinone metabolism were observed, suggesting potential microbiome perturbations and metabolic pathway disruptions [46,47]. The metabolic pathways associated with carbohydrate metabolism were significantly elevated in the gut microbiota of healthy individuals compared to the overweight or obese group, similar to findings reported in individuals with obesity and metabolic syndrome [48]. However, the PICRUSt analysis is a computational approach that uses 16S rRNA sequencing data to predict the functional gene content of microbial communities. It is important to approach the results of this analysis with caution, as they are predictions rather than definitive measurements of gene activity. Further experimental validation, such as metagenomic sequencing or metatranscriptomic analysis, is necessary to confirm the actual metabolic activity of the predicted pathways within the microbial community. This validation step is essential for gaining a more comprehensive understanding of the functional potential and activity of the microbial community.

This study provides insightful findings on the correlation between gut microbiota and obesity, particularly concerning inflammation and dietary factors. Despite its strengths, this study has some limitations. As a cross-sectional study, it presents difficulties in establishing causal relationships between the observed associations. To address this, longitudinal studies are vital for pinpointing whether changes in gut microbiota are the cause or consequence of obesity and inflammation. Additionally, the relatively small sample size of the healthy group may produce clinically relevant results, but it could limit statistical power. An extensive investigation involving a substantial healthy subject sample size would augment reliability through variance reduction and the augmentation of statistically substantial findings. Notably, this study was conducted exclusively in the Thai population, so its findings may not be generally applicable. Lastly, the lack of measurement of SCFA metabolites in this study may result in an incomplete explanation of the relationship between gut microbiota and clinically relevant factors.

## 5. Conclusions

Our study has revealed a substantial divergence in the gut microbiota profile between participants who were or were not overweight or obese. Specifically, the overweight or obese group demonstrated a marked decrease in their microbial diversity and distinct alterations in their gut microbiota composition. *Prevotella* was notably enriched in the overweight or obese cohort, consistent with prior research. Our findings strongly indicate a potential pathway of LPS biosynthesis in obesity, with elevated levels of LPS—linked to a high-fat diet—contributing to localized intestinal inflammation, systemic pro-inflammatory cascades, and triggered insulin resistance. Further comprehensive studies utilizing proteomic or metabolomic approaches are essential to unravel the intricate mechanisms underlying the microbiome’s influence on metabolism in individuals with obesity, which could help prevent metabolic disorders.

## Figures and Tables

**Table 1:** Characteristics of the study population categorized by BMI.

	Healthy Group (*n* = 20)	Overweight/Obese Group (*n* = 75)	*p*-Value
Age, year	38.00 (35.00–50.00)	41.00 (35.00–50.00)	0.024
BMI kg/m^2^	21.10 (19.10–22.80)	27.30 (23.10–42.00)	0.000
Waist circumference, cm	75.01 (63.50–89.50)	92.00 (74.20–116.40)	0.000
Hip circumference, cm	94.50 (87.00–101.10)	106.03 (93.20–132.70)	0.000
Waist–hip ratio	0.80 (0.72–0.89)	0.86 (0.76–0.98)	0.000
Fat mass, kg	15.05 (10.10–18.10)	26.45 (18.80–58.10)	0.000
Body fat, %	28.20 (22.30–31.40)	38.14 (31.30–56.80)	0.001
SBP, mmHg	115.00 (104.00–128.00)	133.00 (102.00–165.00)	0.002
DBP, mmHg	71.00 (59.00–82.00)	82.00 (69.00–120.00)	0.001
Total cholesterol, mg/dL	188.00 (142.00–204.00)	231.00 (151.00–307.00)	0.024
Triglyceride, mg/dL	75.04 (50.32–142.32)	121.06 (66.36–232.19)	0.005
LDL-C, mg/dL	128.32 (67.01–123.54)	153.29 (96.87–232.56)	0.002
HDL-C, mg/dL	65.02 (45.67–84.14)	52.47 (32.51–97.26)	0.000
FPG, mg/dL	82.09 (69.18–95.28)	103.47 (72.00–168.44)	0.003
HbA1C, %	5.10 (4.60–5.70)	8.50 (4.70–8.69)	0.000
Insulin, µU/mL	4.40 (3.20–9.10)	8.45 (3.70–36.10)	0.000
HOMA-IR	0.54 (0.39–0.94)	1.11 (0.56–3.51)	0.001
BUN,	9.90 (6.60–16.60)	10.65 (7.00–15.70)	0.891
Creatinine,	0.69 (0.51–0.89)	0.69 (0.44–0.98)	0.534
AST	23.00 (16.00–28.00)	22.00 (14.00–58.00)	0.402
ALT	15.00 (7.00–26.00)	21.00 (7.00–65.00)	0.000
hs-CRP, mg/L	0.57 (0.40–3.48)	3.37 (0.48–11.24)	0.000
IL-6, pg/mL	2.74 (2.51–3.66)	4.69 (4.03–6.04)	0.000
TNF-alpha, pg/mL	11.34 (10.20–14.15)	15.55 (13.94–19.31)	0.000

**Table 2:** Energy, macronutrients, and micronutrients intake per day of study population.

	Healthy Group (*n* = 20)	Overweight /Obese Group (*n* = 75)	*p*-Value
Total calories, kcal	1531.00 (1179.00–1979.00)	1417.00 (623.00–2298.00)	0.563
Carbohydrate, g	175.00 (153.00–250.00)	205.34 (127.00–375.00)	0.005
Energy from carbohydrates (%)	35.04 (23.04–69.06)	52.33 (26.32–69.39)	0.000
Protein, g/day	54.34 (31.64–100.63)	66.37 (24.60–144.33)	0.058
Energy from protein (%)	14.72 (8.71–33.32)	18.72 (9.05–28.33)	0.028
Protein—Animal, g	35.21 (23.79–85.78)	53.67 (22.00–117.90)	0.008
Protein—Vegetable, g	16.40 (10.85–23.73)	12.16 (10.04–96.55)	0.103
Fat, g	78.77 (34.69–102.24)	49.28 (24.60–144.33)	0.001
Energy from fat (%)	43.53 (21.69–50.56)	29.08 (17.65–50.46)	0.000
Cholesterol, mg	228.00 (48.00–773.00)	339.00 (56.00–1630.00)	0.000
Fiber, g	10.38 (5.78–39.29)	11.16 (5.18–35.40)	0.579
Calcium, mg	225.00 (138.00–665.00)	324.00 (142.00–956.00)	0.005
Phosphorus, mg	628.00 (465.00–1070.00)	584.00 (481.00–1123.00)	0.963
Iron, mg	9.15 (5.02–38.00)	11.08 (5.02–55.13)	0.279
Iron—Animal, mg	2.61 (2.01–11.17)	4.45 (2.05–27.07)	0.044
Iron—Vegetable, mg	5.00 (2.13–8.61)	5.08 (1.70–53.28)	0.263
Potassium, mg	1611.00 (525.00–3177.00)	1773.00 (819.00–3539.00)	0.588
Sodium, mg	1946.00 (1379.00–3979.00)	2445.00 (1202.00–4119.00)	0.021
Copper, mg	0.70 (0.42–1.70)	0.74 (0.42–4.68)	0.266
Magnesium, mg	95.14 (12.98–259.83)	43.71 (10.80–165.50)	0.013
Selenium, mcg	37.57 (10.32–61.56)	31.37 (5.01–120.72)	0.544
Zinc, mg	2.69 (2.02–8.10)	4.51 (2.13–8.28)	0.037
Vitamin A, RAE	301.00 (262.00–1923.00)	321.00 (210.00–3437.00)	0.137
Retinol, µg	140.00 (88.00–1887.00)	240.00 (29.00–2967.00)	0.088
Beta-Carotene, µg	1826.00 (252.00–4372.00)	530.00 (141.00–3787.00)	0.038
Thiamin-B1, mg	1.38 (1.02–1.99)	1.19 (1.09–14.72)	0.335
Riboflavin-B2, mg	0.93 (0.71–3.42)	1.17 (0.58–8.36)	0.051
Vitamin-B6, mg	0.78 (0.16–1.13)	0.53 (0.14–1.19)	0.087
Niacin, mg	13.31 (7.37–18.42)	11.26 (5.77–28.32)	0.66
Vitamin-B12, µg	0.59 (0.40–3.21)	0.83 (0.14–19.94)	0.877
Vitamin C, mg	65.12 (14.46–450.65)	62.09 (9.59–488.22)	0.686
Vitamin E, mg	0.87 (0.39–32.14)	1.19 (0.38–6.84)	0.074

Data were expressed as median (min–max); comparison between two groups by Mann Whitney–U test. RAE: retinol activity equivalents.

**Table 3:** The correlation between blood biochemistry and inflammatory markers of total participants (*n* = 95).

Variable	hs-CRP		IL-6		TNF-α	
	r	*p*-Value	r	*p*-Value	r	*p*-Value
Age	0.142	0.183	0.128	0.268	0.192	0.085
SBP (mmHg)	0.350	0.001	0.335	0.003	0.250	0.025
DBP (mmHg)	0.249	0.018	0.358	0.001	0.264	0.018
BMI (kg/m^2^)	0.632	0.000	0.467	0.000	0.402	0.000
WC (cm)	0.548	0.000	0.377	0.001	0.376	0.001
HC (cm)	0.575	0.000	0.434	0.000	0.422	0.000
Waist–hip ratio	0.306	0.003	0.142	0.216	0.166	0.140
Fat (%)	0.638	0.000	0.526	0.000	0.476	0.000
Fat mass(kg)	0.613	0.000	0.459	0.000	0.395	0.000
Total cholesterol (mg/dL)	0.252	0.017	0.222	0.052	0.288	0.009
Triglyceride (mg/dL)	0.306	0.004	0.205	0.077	0.253	0.023
HDL-Cholesterol (mg/dL)	−0.410	0.000	−0.203	0.076	−0.124	0.271
LDL-Cholesterol (mg/dL)	0.418	0.000	0.289	0.011	0.249	0.025
FPG (mg/dL)	0.275	0.010	0.064	0.582	0.091	0.426
HbA1C (%)	0.132	0.218	0.090	0.438	0.174	0.121
Insulin (uU/mL)	0.451	0.000	0.360	0.001	0.240	0.033
HOMA-IR	0.348	0.001	0.304	0.008	0.244	0.029
AST (U/L)	0.338	0.001	0.055	0.635	0.107	0.210
ALT (U/L)	0.462	0.000	0.156	0.179	0.195	0.083
Creatinine (mg/dL)	−0.095	0.378	−0.021	0.859	0.059	0.598
BUN (mg/dL)	0.049	0.647	−0.010	0.931	−0.052	0.642
Total calories, kcal	0.388	0.000	0.058	0.622	0.098	0.386
Carbohydrate, g/day	0.124	0.667	0.109	0.486	0.108	0.342
Energy from carbohydrates (%)	0.097	0.215	0.118	0.078	0.098	0.094
Protein, g/day	−0.026	0.811	−0.075	0.521	0.109	0.336
Energy from protein (%)	0.121	0.081	0.109	0.124	0.209	0.106
Fat, g/day	0.248	0.020	0.210	0.069	0.173	0.125
Energy from fat (%)	0.368	0.014	0.249	0.006	0.261	0.029
Cholesterol, mg	0.317	0.007	0.127	0.097	0.171	0.109

**Table 4:** The gut microbial composition (relative abundance in %) of overweight/obese and healthy subjects (phylum level).

Phylum	Healthy (%)	Overweight/Obese (%)	*p*-Value	q-Value
Firmicutes	55.60	52.50	0.2938	0.4433
Bacteroidota	22.40	34.60	9.17 × 10^−5^	0.0004
Actinobacteriota	17.00	7.30	1.64 × 10^−5^	0.0001
Proteobacteria	3.50	3.80	0.2855	0.4433
Verrucomicrobiota	0.70	0.20	0.0528	0.1585
Fusobacteriota	0.60	1.30	0.3210	0.4433
Desulfobacterota	0.30	0.40	0.8802	0.8802
F–B ratio	2.80	1.96	0.0351	0.0012

**Table 5:** The difference in bacterial composition between overweight/obese and healthy subjects (phylum and genus levels).

Genus	Phylum	Log2FC	*p*-Value
More prevalent in healthy subjects			
Acidaminococcus	Firmicutes	−4.040	0.017
Coprobacillus	Firmicutes	−3.400	0.039
Lactobacillus	Firmicutes	−2.650	0.000
CAG_352	Firmicutes	−1.880	0.000
Bifidobacterium	Actinomycetota	−1.500	0.003
Akkermansia	Verrucomicrobiota	−1.450	0.000
Collinsella	Actinomycetota	−0.605	0.037
Megasphaera	Firmicutes	−0.442	0.000
Senegalimassilia	Actinomycetota	−0.402	0.000
Eubacterium_ruminantium_group	Firmicutes	−0.398	0.000
Faecalibacterium	Firmicutes	−0.056	0.000
More prevalent in overweight/obese subjects			
Megamonas	Firmicutes	0.213	0.000
Sutterella	Pseudomonadota	0.305	0.000
Dialister	Firmicutes	0.356	0.000
Holdemanella	Firmicutes	0.454	0.000
Lachnospiraceae_UCG_008	Firmicutes	0.459	0.000
Butyricicoccus	Firmicutes	0.517	0.015
Bacteroides	Bacteroidota	0.636	0.007
Prevotella	Bacteroidota	0.898	0.000
Lachnospiraceae_UCG_004	Firmicutes	1.040	0.001
Lachnoclostridium	Firmicutes	1.330	0.000
Sellimonas	Firmicutes	1.490	0.000
Lactococcus	Firmicutes	1.800	0.025
Ruminococcus_gnavus_group	Firmicutes	2.100	0.030

## Data Availability

The original contributions presented in the study are included in the article, further inquiries can be directed to the corresponding author. Data pertaining to the microbiota analysis are accessible in the repository at https://dataview.ncbi.nlm.nih.gov/object/PRJNA1128428?reviewer=hj2o1gd224u9k0m6kk3meghcd5, accessed on 27 June 2024.
